# Factors associated with genetic testing distress in patients tested for Lynch Syndrome or Hereditary Breast and Ovarian Cancer Syndrome

**DOI:** 10.1186/1897-4287-9-S1-P33

**Published:** 2011-03-10

**Authors:** Margery Rosenblatt, Monica Dandapani, Rowena Mercado, Judy E Garber, Sapna Syngal, Elena M Stoffel

**Affiliations:** 1 Dana-Farber Cancer Institute, 44 Binney Street, Boston, MA 02115, USA

## Background

The emotional impact of genetic testing has been extensively studied in individuals at high risk for Hereditary Breast and Ovarian Cancer Syndrome (HBOS). Studies on the impact of genetic testing for Lynch Syndrome (LS) are not as prevalent and few studies have compared the two cohorts.

## Purpose

To compare the psychological impact of genetic testing in participants at high risk for LS and HBOS and identify variables associated with genetic testing distress, anxiety and depression at one week and one year post disclosure of genetic test results.

## Methods

140 individuals undergoing genetic testing for LS and 133 undergoing genetic testing for HBOS at the Dana-Farber Cancer Institute were enrolled in a longitudinal questionnaire study. We assessed levels of genetic testing distress, anxiety and depression using The Multidimensional Impact of Cancer Risk Assessment (MICRA) and the Hospital Anxiety and Depression Scale (HADS). Multivariable regression analysis was used to identify factors associated with psychological distress in the LS and HBOS cohorts. One year follow-up data is based on 96 LS and 94 HBOS tested individuals that completed the one year questionnaire.

## Results

For both cohorts, individuals who had the expectation for carrying a deleterious gene pre-test and/or those who received a positive or variant of uncertain significance (VUCS) test result had significantly higher genetic testing distress at one week post-disclosure (p ≤ 0.05). Additional independent predictors of higher genetic testing distress in the LS cohort included not being married and having a personal history of cancer. In the HBOS cohort, predictors included having a higher HADS anxiety score at baseline and having an annual household income of ≤ $50,000.

At one year post-disclosure, individuals that tested positive or VUCS and/or had the expectation of carrying a deleterious gene pre-test continued to have significantly higher genetic testing distress (p ≤ 0.01). While there were no differences between the cohorts in baseline or post-disclosure HADS anxiety or depression scores, significantly more individuals in the LS cohort had HADS depression scores ≥ 8 one year after testing (17% vs. 5%; p= 0.05).

## Conclusion

Individuals at risk for LS and HBOS whose genetic tests reveal a pathogenic mutation or VUCS can continue to demonstrate genetic testing distress one year after receiving genetic test results. These individuals may benefit from counseling beyond the immediate disclosure period.

